# A comparison of the persuasiveness of human and ChatGPT generated pro-vaccine messages for HPV

**DOI:** 10.3389/fpubh.2024.1515871

**Published:** 2025-01-16

**Authors:** Dengke Xia, Mengyao Song, Tingshao Zhu

**Affiliations:** ^1^Department of Psychology, Shandong Second Medical University, Weifang, China; ^2^CAS Key Laboratory of Behavioral Science, Institute of Psychology, Chinese Academy of Sciences, Beijing, China; ^3^Department of Psychology, University of Chinese Academy of Sciences, Beijing, China

**Keywords:** large language models, AI-mediated communication, persuasion, public health messages, message factors

## Abstract

**Introduction:**

Public health messaging is crucial for promoting beneficial health outcomes, and the latest advancements in artificial intelligence offer new opportunities in this field. This study aimed to evaluate the effectiveness of ChatGPT-4 in generating pro-vaccine messages on different topics for Human Papillomavirus (HPV) vaccination.

**Methods:**

In this study (*N* = 60), we examined the persuasive effect of pro-vaccine messages generated by GPT-4 and humans, which were constructed based on 17 factors impacting HPV vaccination. Paired-samples t-tests were used to compare the persuasiveness of these messages.

**Results:**

GPT-generated messages were reported as more persuasive than human-generated messages on some influencing factors (e.g., *untoward effect*, *stigmatized perception*). Human-generated messages performed better on the message regarding convenience of vaccination.

**Discussion:**

This study provides evidence for the viability of ChatGPT, in generating persuasive pro-vaccine messages to influence people’s vaccine attitudes. It is indicated that the feasibility and efficiency of using AI for public health communication.

## Introduction

Public health messaging is critical in promoting beneficial public health outcomes. Effective public health campaigns hold significant societal importance, as they nudge people to make better decisions for themselves and their communities (1, 2). Public health messaging research focuses on composing messages that deliver crucial information to remind individuals to adopt behaviors conducive to health (3).

In recent years, Artificial Intelligence (AI) has made significant achievement for generating information. Previous research has explored AI’s impact on health information-seeking behaviors (4), as well as the ethical implications of AI and data in public health communication and persuasion (5, 6). With the development of Large Language Models (LLMs), it has demonstrated powerful generative capabilities, and has achieved notable success in content creation across various contexts, including the public health domain (7, 8). ChatGPT developed by OpenAI is one of such LLMs. Within just two months of its launch, it surpassed 100 million users and reached over 1.5 billion monthly visits. It is necessary to explore the applications of ChatGPT in public health messaging.

Currently, the potential benefit to use LLMs in vaccination has been reported. Deiana et al. (9) found that ChatGPT can provide accurate information in a conversational way, addressing common myths and misconceptions about vaccination (9). Karinshak et al. (8) conducted a systematic evaluation of GPT-3’s ability to generate COVID-19 pro-vaccine messages, found that AI can create effective public health messages under human supervision (8). In addition, Kim et al. (10) found that ChatGPT can utilize content from social media to analyze public opinions on vaccination (10).

Cervical cancer, as one of the major global health concerns, poses a significant threat to the public health (11). Despite getting vaccinated against HPV (Human Papillomavirus) is an important method to prevent cervical cancer (12), HPV vaccination hesitancy and low vaccination rates still persist. A systematic review suggests that only 15–31% of respondents have even heard of HPV, while only 0.6–11% know that HPV is a risk factor for cervical cancer (13). Moreover, the abundance of misinformation about vaccination on the internet has led to a decline in public acceptance and trust in vaccinations, presenting significant challenges for the promotion of HPV vaccination (14, 15).

Currently, less researches have been conducted for evaluating GPT-4’s performance to generate pro-vaccine messages for HPV. Although prior studies have shown that AI can support human decision-making and persuasion (16, 17), including in communication tasks within high risk areas such as public health (18, 19), the generation capability of pro-vaccine messages on different topics remains unclear. Previous research has identified 17 influencing factors that individuals may take into account when deciding whether to get HPV vaccine (20), providing an ideal framework for generating HPV vaccination information. In this study, we use this framework to construct pro-vaccine messages according to these influencing factors, and explore the differences in persuasiveness between ChatGPT and human generated HPV pro-vaccine messages. We propose the following hypothesis:

*H:* ChatGPT generated HPV pro-vaccine messages are more persuasive than human on some influencing factors.

## Methods

### Participants

According to G⁎Power, we would need 45 participants when conducting paired samples t-test. In this study, a total of 60 undergraduate and graduate students were finally recruited, with an average age of 23.03 (*SD* ± 2.22). Our study has received ethical approval from the Institutional Review Board of the Institute of Psychology, Chinese Academy of Sciences with the ethics approval number H23089. All participants in the study were adults and signed a consent form after being fully informed.

### Materials

**Figure 1:**
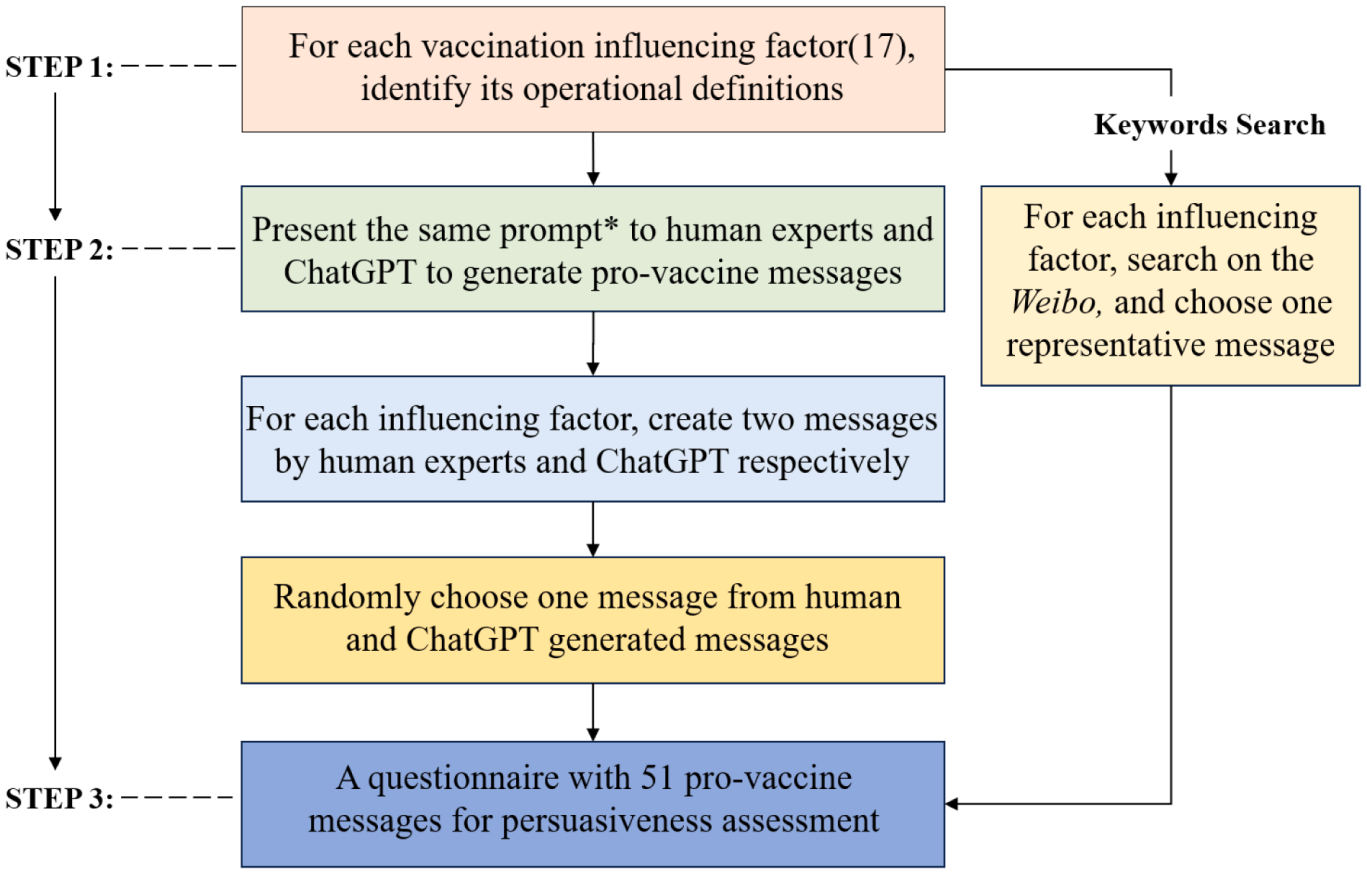
The process of questionnaire development. *The prompt used in this study: “Please craft a persuasive vaccine message, based on the following influencing factor and its operational definitions, to encourage individuals to get the HPV vaccine, in no more than 100 words”.

A questionnaire was developed in this study to measure the persuasiveness of messages generated according to 17 influencing factors. These factors were derived from a study by Song and colleagues on determinants of HPV vaccination decision among young Chinese women (20). Through semi-structured interviews, they identified 17 key factors influencing audiences’ vaccination decisions (e.g., *vaccine safet*y, *untoward effect*) (see Table 1). For each message, we added the item measuring the perceived persuasiveness, “I think this message is persuasive in promoting the HPV vaccination” (1 = *Strongly disagree*, 7 = *Strongly agree*). The whole procedure is depicted in Figure 1.

**Table 1:** The name and definition of each influencing factor.

No.	Influencing factor	Operational definition
1	Vaccine safety	The vaccine does not pose a threat to personal safety, such as a low incidence rate of serious untoward effect and the good health condition of population after vaccination.
2	Vaccination restriction and contraindication	The range of specific people who are not suitable for vaccination or contraindications.
3	Untoward effect	A clear and unambiguous description of the specifics of the adverse reaction and does not contain arbitrary, omitted or ambiguous expressions.
4	Convenience of vaccination	The costs and considerations of the individual vaccination process, such as distance, time, and the vaccination process.
5	Official position	The power of public trust in public authority
6	Unofficial position	The publisher of the message is not the official medium, but some media with a neutral political position and no political purpose.
7	Scientific principle	Accessible and accurate scientific message about vaccines presented in clear and understandable language for the public.
8	Dual role persuasion	Both positive and negative aspects are provided in the message.
9	High standard group	The message reflects the use of other out-groups as references when individuals adopt a certain behavior or form a certain attitude. These groups usually have the value of benchmarking and imitation.
10	Surrounding group	Individual takes a certain behavior; it is influenced by the people around the individual or who have the same background as the individual’s life.
11	Conformity	Vaccination and the behaviors and perceptions that promote it are the choice of most people, and are something that people are generally willing to do.
12	Provide room for choice	Provide various qualities and effects of goods for users to compare and choose.
13	Scarcity	Reflect the scarcity or unavailability of vaccines in the message.
14	Data	Provide specific numerical values in the message
15	Vicarious experience	Individuals are able to gain awareness of vaccination-related content by observing the behavior of others
16	Stigmatized Perception	Misconceptions about Vaccinations
17	Interpretability-External attribution	Explanations in the message for the relevant behaviors and requirements in the process of vaccine development, vaccination, and promotion, such as the reasons for the specific unsuitability of the unsuitable population for vaccination.

### Data collection and analysis

All experimental procedures were conducted with the informed consent of the participants. Participants read the pro-vaccine messages at first, which were presented in a randomized order, and reported their perceived persuasiveness. Then, to examine differences in the persuasiveness of human-authored, GPT-generated pro-vaccine messages and baseline messages, we used paired-samples t-tests. All of these analyses were conducted using SPSS 27.

## Results

The analysis of the persuasiveness reported in Table 2 shows that differences between the GPT-generated, human-generated and baseline messages are significant on several influencing factors. Specifically, the persuasiveness of GPT-generated messages was significantly higher for the messages about negative cue (e.g., *untoward effect*, *stigmatized perception*) and group behavior (e.g., *high standard group*, *conformity*) compared to human-authored. This indicates that GPT-generated messages are more persuasive compared to human-authored ones. The persuasiveness of the practical information (e.g., *convenience of vaccination*) messages generated by GPT-4 was significantly lower than human-authored. It suggests that the messages constructed based on *convenience of vaccination*, human-generated messages are more persuasive than GPT-generated. In addition, the results of comparing of the GPT-generated and human-generated messages with baseline messages shows that both GPT and human generated messages were more persuasive than the baseline messages on most influencing factors. However, for messages related to the objective (e.g., *unofficial position*, *scientific principle* and *dual role persuasion*), the baseline messages were significantly more persuasive than both GPT and human generated.

**Table 2:** The persuasiveness comparison of human-generated messages, GPT-generated messages and baseline messages.

Influencing factors	Human vs. GPT-4		Human vs. Baseline		GPT-4 vs. Baseline	
	*t*	*p*	*t*	*p*	*t*	*p*
Vaccine safety	−1.762	0.083	6.554	<0.001	7.349	<0.001
Vaccination restriction and contraindication	1.951	0.056	5.655	<0.001	4.22	<0.001
Untoward effect	−3.22	0.002	3.098	0.003	4.667	<0.001
Convenience of vaccination	2.151	0.036	3.472	0.001	2.03	0.047
Official position	0.875	0.385	3.891	<0.001	2.508	0.015
Unofficial position	−0.095	0.925	−4.752	<0.001	−3.621	0.001
Scientific principle	−0.522	0.604	−2.449	0.017	−2.382	0.02
Dual role persuasion	−0.452	0.653	−2.92	0.005	−2.068	0.043
High standard group	−3.094	0.003	0.091	0.928	2.949	0.005
Surrounding group	1.574	0.121	7.681	<0.001	6.047	<0.001
Conformity	−3.9	<0.001	1.105	0.274	3.246	0.002
Provide room for choice	−1.762	0.083	0.333	0.74	1.513	0.136
Scarcity	−0.351	0.727	0.993	0.325	1.101	0.275
Data	1.013	0.315	−0.198	0.843	−1.277	0.207
Vicarious experience	1.758	0.084	4.633	<0.001	3.191	0.002
Stigmatized perception	−3.834	<0.001	0.721	0.474	3.4	0.001
Interpretability-external attribution	0.599	0.552	1.932	0.058	1.386	0.171

## Discussion

This study compared the performance of creating persuasive pro-vaccine messages by 17 influencing factors between GPT-4 and human-authored. The results showed that GPT-generated messages regarding *untoward effect*, *stigmatizing perception*, *high-standard group*, and *conformity* were significantly more persuasive. This study highlights the potential value of using LLMs in public health communication and demonstrates that models such as GPT-4 can be useful tools for augmenting content generation.

GPT-4 outperformed humans in decreasing public concern about vaccine side effects and correcting misconceptions about HPV vaccines. Vaccine side effects are always a crucial issue of public concern (21), while stigmatized views of HPV also contribute to vaccine hesitancy (22). Prior study showed that ChatGPT generates longer and more diverse responses when given prompts containing negative content (23). For HPV vaccination, The negative cues (e.g., *untoward effect*, *stigmatized perception*) prompt GPT generate more detailed explanations, enhancing the messages’ persuasiveness. Although GPT has exhibited limitations concerning the interpretation of cultural context (24), since GPT-4 was trained on trillions of words from the internet, it can capture the characteristics of public language and generate messages that align with social norms and public perceptions (7, 25). As a result, GPT-generated messages showed greater persuasiveness regarding the messages about group behavior (e.g., *high standard group*, *conformity*) too.

In addition, GPT-generated *convenience of vaccination* messages showed lower persuasive than human generated. Since ChatGPT uses different linguistic devices than humans (26),and AI’s language strategies may not align with human preferences in specific areas. Readers may expect concise and relatable language when discussing the convenience of vaccination. GPT-4 too formal or lack contextual adaptability (27), which diminishes the emotional connection and persuasiveness of the messages.

However, the persuasiveness of pro-vaccine messages may be reduced when they have an obvious persuasive intent on certain topics. In our study, some messages (e.g., *unofficial position*, *dual role persuasion* and *scientific principle*) require authenticity and objectivity of viewpoint. If we construct such messages around a specific purpose, they may appear too deliberate and official, which diminishes public trust.

Our findings could bring some inspirations to the persuading practices in vaccination promotion or even broader field. The present results provide evidence for the viability of LLMs, particularly ChatGPT, in generating persuasive pro-vaccine messages to influence people’s vaccine attitudes. GPT-4’s performance in comparison to human-authored content reveals that LLMs have the potential to support public health program. With the unique ability to draw upon massive amounts of textual data, LLMs are able to synthesize extensive, diverse sources in content generation almost instantly in contrast to existing workflows, which require extensive, expensive consumer research processes. GPT-generated messages will lead to higher levels of persuasiveness and better express the benefits of vaccination on certain topics (e.g., *untoward effect*); thus, they will promote the intention to get the COVID-19 vaccine. Although prompt development can take a significant amount of time, once effective prompts have been identified, GPT-4 can easily generate a large number of unique messages. Our results suggests that AI has the potential to contribute to public health communication workflows to effectively and efficiently develop strategic communication campaigns. In addition, we intentionally used structured prompts, such structured prompts can still serve as a useful reference for human experts, helping them generate more effective messages.

The current study also has several limitations. First, our study was conducted in China, and the results might be applicable in China only. People should use the results of this study with caution in the context of different cultural backgrounds. Our study focused on the perspective of undergraduate and graduate. Little is known about whether these results found in this study would work well for the other groups. Future study should further investigate on difference population. In addition, the primary focus of this experiment was to examine the persuasiveness of the messages, but one single question may not be sufficient to fully assess their impact, and it is ideal to examine the audiences’ behavioral changes (e.g., willingness to vaccinate) after exposure to specific types of information.

## Conclusion

The results of this study suggest that GPT-4 has the potential to effectively generate persuasive pro-vaccine messages, such as HPV vaccination. GPT-generated pro-vaccine messages constructed around *untoward effect*, *stigmatizing perception*, *high-standard group*, and *conformity* showed significantly higher persuasiveness than human generated. But the *convenience of vaccination* message was lower. It suggests that GPT-4 can be adopted to mediate public health messaging by quickly generating message drafts and the appropriate call to action, with human oversight. This study demonstrates the feasibility and efficiency of using LLMs for public health communication, contributing to the field of AI application.

## Data Availability

The original contributions presented in the study are included in the article/supplementary material, further inquiries can be directed to the corresponding author.
